# Temporal and spatial trends in marine carbon isotopes in the Arctic Ocean and implications for food web studies

**DOI:** 10.1111/gcb.14832

**Published:** 2019-10-10

**Authors:** Camille de la Vega, Rachel M. Jeffreys, Robyn Tuerena, Raja Ganeshram, Claire Mahaffey

**Affiliations:** ^1^ School of Environmental Sciences University of Liverpool Liverpool UK; ^2^ School of Geosciences University of Edinburgh Edinburgh UK

**Keywords:** base of the food web, dissolved inorganic carbon, isoscape, marine mammals, particulate organic matter, sea ice decline, Suess effect, δ^13^C

## Abstract

The Arctic is undergoing unprecedented environmental change. Rapid warming, decline in sea ice extent, increase in riverine input, ocean acidification and changes in primary productivity are creating a crucible for multiple concurrent environmental stressors, with unknown consequences for the entire arctic ecosystem. Here, we synthesized 30 years of data on the stable carbon isotope (δ^13^C) signatures in dissolved inorganic carbon (δ^13^C‐DIC; 1977–2014), marine and riverine particulate organic carbon (δ^13^C‐POC; 1986–2013) and tissues of marine mammals in the Arctic. δ^13^C values in consumers can change as a result of environmentally driven variation in the δ^13^C values at the base of the food web or alteration in the trophic structure, thus providing a method to assess the sensitivity of food webs to environmental change. Our synthesis reveals a spatially heterogeneous and temporally evolving δ^13^C baseline, with spatial gradients in the δ^13^C‐POC values between arctic shelves and arctic basins likely driven by differences in productivity and riverine and coastal influence. We report a decline in δ^13^C‐DIC values (−0.011‰ per year) in the Arctic, reflecting increasing anthropogenic carbon dioxide (CO_2_) in the Arctic Ocean (i.e. Suess effect), which is larger than predicted. The larger decline in δ^13^C‐POC values and δ^13^C in arctic marine mammals reflects the anthropogenic CO_2_ signal as well as the influence of a changing arctic environment. Combining the influence of changing sea ice conditions and isotopic fractionation by phytoplankton, we explain the decadal decline in δ^13^C‐POC values in the Arctic Ocean and partially explain the δ^13^C values in marine mammals with consideration of time‐varying integration of δ^13^C values. The response of the arctic ecosystem to ongoing environmental change is stronger than we would predict theoretically, which has tremendous implications for the study of food webs in the rapidly changing Arctic Ocean.

## Graphical abstract

Warming of the Arctic has led to changes in multiple environmental factors that control the carbon isotope ratio at the base of the food web. Here, we present the first comprehensive spatial and temporal assessment of the carbon isotopic baseline for the Arctic Ocean, which is essential for understanding and predicting changes in Arctic food web structure. The response of the Arctic ecosystem to ongoing environmental change presented here is stronger than we would predict theoretically.

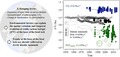

## 1 INTRODUCTION

The Arctic is changing rapidly (IPCC, 2013), warming twice as fast as the global average (Carmack et al., 2015; Hoegh‐Guldberg & Bruno, 2010) and causing sea ice to decline in both extent and thickness (Kwok, 2018; Lind, Ingvaldsen, & Furevik, 2018). Sea ice underpins the entire arctic ecosystem and the decline in this seasonal habitat is affecting the entire food web. Primary production has increased by 30% from 1998 to 2012 owing to an increase in light under reduced ice conditions (Arrigo & van Dijken, 2015). Arctic predators, such as seals and polar bears, that rely on sea ice for foraging, moulting and breeding are also adversely affected by the loss of sea ice (Laidre et al., 2008). Other climate‐induced changes are occurring in tandem and include acidification (Yamamoto, Kawamiya, Ishida, Yamanaka, & Watanabe, 2012), shifts in wind patterns and enhanced wind field in the Western Arctic (Overland & Wang, 2010), increased coastal erosion, river flow and melting of permafrost and glaciers (Haine et al., 2015; Jones et al., 2009; Mars & Houseknecht, 2007). These multiple concurrent stressors have far‐reaching implications for the arctic marine ecosystem at multiple trophic levels, and there is an urgent need to understand the ecosystem response in this unique polar habitat.

The ratio of stable carbon isotopes, ^13^C and ^12^C, expressed as δ^13^C (‰), provides a powerful tool for studying food webs. The δ^13^C values of particulate organic carbon (POC), consisting of fresh phytoplankton, microzooplankton, bacteria and marine and terrestrial detritus, (Fry & Sherr, 1989; Lobbes, Fitznar, & Kattner, 2000; Michener & Kaufman, 2007; Wassmann et al., 2004), represent the base of the food web or ‘baseline’. The δ^13^C values of POC (δ^13^C‐POC) are generally transferred with a ^13^C enrichment of 1‰–2‰ between each trophic level, creating an inextricable link between the base of the food web and consumers (Fry, Anderson, Entzeroth, Bird, & Parker, 1984). Spatial trends in δ^13^C‐POC values controlled by environmental factors have been used to decipher the foraging and migratory patterns of consumers on a regional scale (Hoffman, 2016; Iken, Bluhm, & Dunton, 2010; Polito et al., 2017; Wassenaar, 2019) and more recently on a global scale in the construction of global ‘isoscapes’ (Bird et al., 2018; Bowen & West, 2008; Firmin, 2016; Graham, Koch, Newsome, McMahon, & Aurioles, 2010; McMahon, Hamady, & Thorrold, 2013b). However, spatial and temporal trends in the δ^13^C values of high trophic levels may also reflect changes in food web structure such as loss or addition of species, consumer's diet or a combination of factors. To disentangle the drivers of spatial and temporal trends in the δ^13^C values of consumers in the Arctic, it is crucial to establish spatial and temporal variations in δ^13^C values at the base of the food web, allowing the sensitivity of marine arctic consumers to environmental change to be quantified.

It is challenging to isolate phytoplankton‐POC for analysis and so the nominal definition of δ^13^C‐POC values typically assumes that the bulk of POC is derived from phytoplankton only, although δ^13^C‐POC values can be influenced by other factors such as bacterial activity and detritus (Michener & Kaufman, 2007). While the detrital fraction of POC may be degraded by bacteria, potentially altering the δ^13^C values of that fraction, we assume that photosynthetic phytoplankton are responsible for transforming the bulk of δ^13^C‐POC values in time and space. δ^13^C value of phytoplankton, which underpins the δ^13^C‐POC values, is controlled by fractionation during photosynthesis. This equates to the difference between the δ^13^C values of the carbon source, either dissolved inorganic carbon (DIC) or carbon dioxide (CO_2_), and the δ^13^C‐POC values (Cassar, Laws, Bidigare, & Popp, 2004; Young, Bruggeman, Rickaby, Erez, & Conte, 2013). Factors such as phytoplankton growth rate, availability or concentration of carbon, light and nutrient availability affect isotopic fractionation and the δ^13^C‐POC values (Burkhardt, Riebesell, & Zondervan, 1999; Keeley & Sandquist, 1992). As such, environmental conditions can create distinct patterns in these values. δ^13^C‐POC values become enriched in ^13^C in an environment where replenishment of the CO_2_ pool is slow or restricted, for example, during periods of rapid phytoplankton growth (Rau, Takahashi, Des Marais, Repeta, & Martin, 1992) or in sea ice associated with sympagic primary production (Budge et al., 2008; Hobson et al., 2002; Søreide et al., 2013; Wang, Budge, Gradinger, Iken, & Wooller, 2014). Conversely, an increase in CO_2_ concentration will lead to a carbon pool depleted in ^13^C (Rau et al., 1992) creating a ^13^C‐deplete POC pool. Terrestrially derived POC delivered via rivers and coastal erosion also tends to be depleted in ^13^C relative to marine‐derived POC (Boutton, 1991; Keeley & Sandquist, 1992). While global isoscapes capture the large‐scale spatial trends in δ^13^C values related to oceanographic provinces (shelf vs. open ocean) and latitude (Bird et al., 2018; Bowen & West, 2008; Graham et al., 2010; McMahon et al., 2013b), they do not include the Arctic Ocean. We expect the δ^13^C values of POC in the Arctic to be influenced by the strong regional trends in sea ice, productivity and terrestrial influence including riverine input and coastal erosion, all of which vary along the water mass circulation pathways from the inflow shelves, which receive water from the Atlantic and Pacific oceans, to the arctic basins and interior shelves (Sakshaug, 2004; Tremblay & Gagnon, 2009; Varela, Crawford, Wrohan, Wyatt, & Carmack, 2013).

Imprinted on the regional trends is a temporal trend in δ^13^C values worldwide. Enhanced atmospheric CO_2_ since the industrial period (Tagliabue & Bopp, 2008) is causing an increase in oceanic CO_2_ (Sabine et al., 2004) and a decline in the δ^13^C values of DIC (δ^13^C‐DIC), known as the Suess effect, as a result of ^13^C‐depleted anthropogenic CO_2_ (Quay, Sonnerup, Westby, Stutsman, & McNichol, 2003). δ^13^C‐DIC values in the Arctic Ocean are predicted to change at a rate of −0.006‰ to −0.008‰ per year, compared to the global average of −0.017‰ per year (Tagliabue & Bopp, 2008). However, several studies have already shown that decadal trends in the δ^13^C values of marine mammals in the Arctic (Misarti, Finney, Maschner, & Wooller, 2009; Nelson, Quakenbush, Mahoney, Taras, & Wooller, 2018; Newsome et al., 2007; Schell, 2001) are larger than the Suess effect alone, implying that other factors are altering their δ^13^C signatures on decadal timescales.

The main objective of this study was to quantify how regional differences and temporal trends in the arctic environment have altered the δ^13^C values in DIC and POC, representing the base of the food web or ‘baseline’. We compared these trends at the base of the food web to trends in δ^13^C values in arctic marine mammals to investigate how environmental change (e.g. Suess effect, loss of sea ice) may alter δ^13^C values in the entire food web. We synthesized published data from 1977 to 2014 on δ^13^C values of DIC and dissolved CO_2_, and δ^13^C‐POC values in the surface ocean (POC_water_) and in sea ice (POC_ice_) across the entire Arctic Ocean, alongside data from arctic rivers (POC_riv_). We quantified regional differences in the δ^13^C values in POC and discuss the underlying environmental drivers of the observed spatial heterogeneity. We then quantified the decadal trends in δ^13^C values of DIC and CO_2_, and δ^13^C values of POC in the Arctic Ocean, comparing the rate of change to the Suess effect and observed trends in tissues of arctic marine mammals from the post‐industrial period.

## 2 MATERIALS AND METHODS

### 2.1 Data collation

Data on bulk δ^13^C‐POC_water_, δ^13^C‐POC_ice_ and δ^13^C‐POC_riv_ values, focusing on suspended particulate organic matter above the thermocline, were collated from tables and figures in 37 original manuscripts and two open access databases for both marine (PANGAEA; http://www.pangaea.de) and riverine (articGRO; https://arcticgreatrivers.org/) environments, in Arctic and sub‐Arctic regions, as defined by the Köppen–Geiger climate classification (Kottek, Grieser, Beck, Rudolf, & Rubel, 2006). The database included 354 data points for marine δ^13^C‐POC_water_ values (Brown et al., 2014; Connelly, McClelland, Crump, Kellogg, & Dunton, 2015; Forest et al., 2010; Griffith et al., 2012; Guo, Tanaka, Wang, Tanaka, & Murata, 2004; Hallanger et al., 2011; Hobson, Ambrose, & Renaud, 1995; Hobson et al., 2002; Iken et al., 2010; Iken, Bluhm, & Gradinger, 2005; Ivanov, Lein, Zakharova, & Savvichev, 2012; Kohlbach et al., 2016; Kuliński, Kędra, Legeżyńska, Gluchowska, & Zaborska, 2014; Kuzyk, Macdonald, Tremblay, & Stern, 2010; Lin et al., 2014; Lovvorn et al., 2005; O'Brien, Macdonald, Melling, & Iseki, 2006; Parsons et al., 1989; Roy et al., 2015; Sarà et al., 2007; Schubert & Calvert, 2001; Smith, Henrichs, & Rho, 2002; Søreide et al., 2008; Søreide, Hop, Carroll, Falk‐Petersen, & Hegseth, 2006; Tamelander, Reigstad, Hop, & Ratkova, 2009; Tamelander et al., 2006; Tremblay, Michel, Hobson, Gosselin, & Price, 2006; Zhang et al., 2012), 69 data points for δ^13^C‐POC_ice_ values (Forest et al., 2010; Hobson et al., 1995; 2002; Iken et al., 2005; Kohlbach et al., 2016; Lovvorn et al., 2005; Roy et al., 2015; Schubert & Calvert, 2001; Søreide et al., 2006, 2008; Tamelander et al., 2006; Tremblay et al., 2006) and 383 data points for riverine δ^13^C‐POC_riv_ values (Goni, Yunker, Macdonald, & Eglinton, 2000; Holmes, McClelland, Tank, Spencer, & Shiklomanov, 2018; Kuzyk et al., 2010; Lobbes et al., 2000). Data were available over different temporal scales: marine δ^13^C‐POC_water_ values from 1986 to 2013, δ^13^C‐POC_ice_ from 1993 to 2012 and riverine δ^13^C‐POC_riv_ values from 1987 to 2016.

To relate the temporal trend in δ^13^C‐POC_water_ values to the predicted decline of δ^13^C‐DIC and δ^13^C‐CO_2_ values, a compilation of data on δ^13^C‐DIC values was extracted from three publications (Bauch, Polyak, & Ortiz, 2015; Schmittner et al., 2013; Young et al., 2013) and two databases (Becker et al., 2016; Key et al., 2015). δ^13^C‐CO_2_ values were determined from the δ^13^C‐DIC values and absolute temperature following the Equation (1) (Rau, Riebesell, & Wolf‐Gladrow, 1996). δ^13^C‐DIC and δ^13^C‐CO_2_ values included 1,333 data points covering 1977–2014.

(1) $$\delta^{13}{\text{C-CO}}_{2}=\delta^{13}\text{C-DIC}+23.644-9,701.5/T,$$

where *T* is the temperature in Kelvin.

To determine if the temporal trend in δ^13^C‐POC values was reflected in higher trophic levels within the Arctic Ocean, δ^13^C data were collated from arctic marine mammals covering years following the industrial period (post 1950). We collated δ^13^C data from teeth of ringed seals (*Pusa hispida*) from 1986 to 2006 from East Greenland (Aubail, Dietz, Rigét, Simon‐Bouhet, & Caurant, 2010) and northern fur seals (*Callorhinus ursinus*) from 1950 to 2000 from the Bering Sea and Gulf of Alaska (Newsome et al., 2007). Additionally, δ^13^C data were collated from teeth of Beluga whales (*Delphinapterus leucas*) from 1963 to 2008 from the Hudson Bay and from 1976 to 2001 from the Baffin Bay (Matthews & Ferguson, 2014), and baleen plates of bowhead whales (*Balaena mysticetus*) from 1950 to 1998 from the Bering and Chukchi Seas (Schell, 2001).

### 2.2 Data treatment

**Figure 1:**
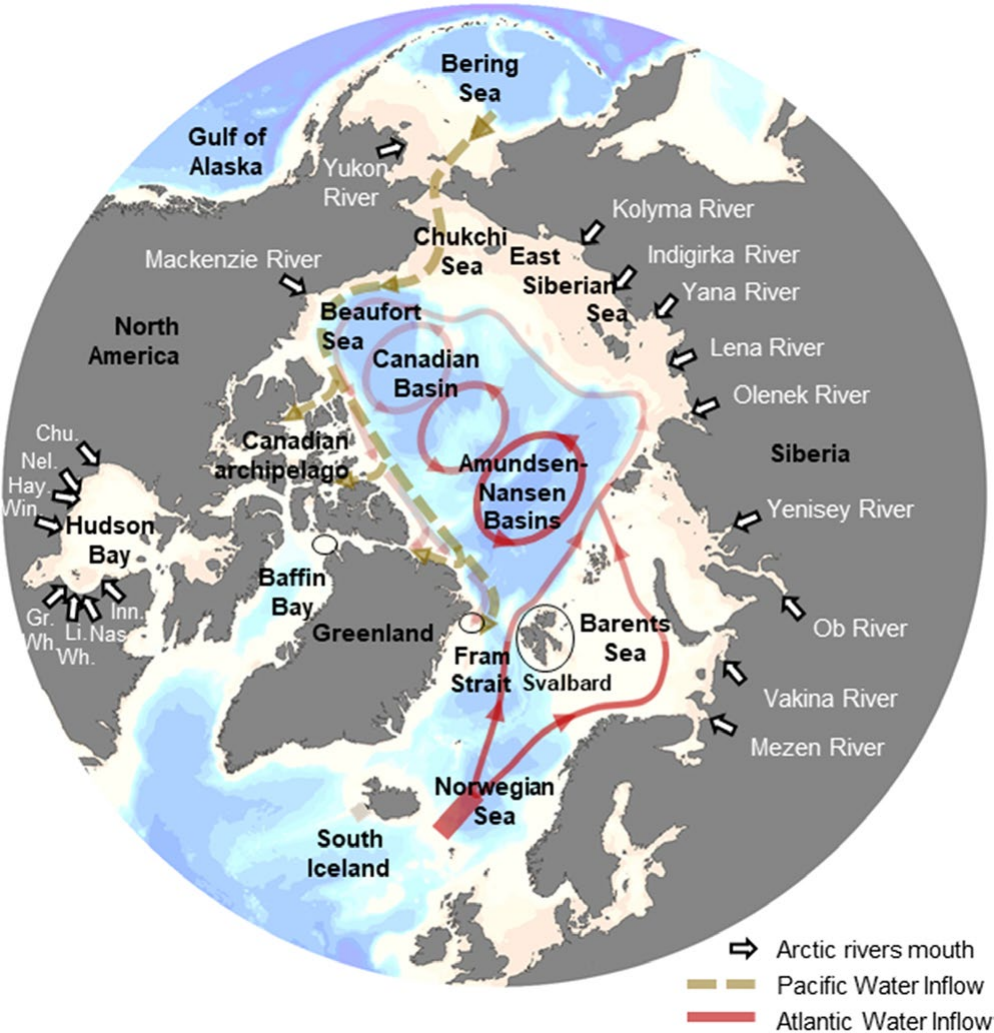
Map indicating the locations of the arctic regions considered in this study. Circulation pathways are highlighted and modified from Carmack and Wassmann (2006). The yellow arrows represent the intermediate Pacific water and the red arrows represent the Atlantic water. White arrows indicate the mouths of the arctic rivers. The black circles point to the approximate location of the North Water Polynia in the Northern Baffin bay, North‐East Water Polynia in Northeast Greenland and Svalbard marine coastal area. Chu., Churchill River; Gr.Wh., Great Whale River; Hay., Hayes River; Inn., Innuksuac River; Li.Wh., Little Whale River; Nas., Nastapoca River; Nel., Nelson River; Win., Winisk River; Bathymetry and coast lines were from the software Ocean Data View (Schlitzer, 2016)

We analysed the δ^13^C‐POC_water_, δ^13^C‐POC_ice_, δ^13^C‐DIC and δ^13^C‐CO_2_ values in 17 marine arctic regions (Figure 1; Table 1). In addition, the δ^13^C‐POC_water_ values from arctic rivers were grouped into two large riverine regions: the Siberian rivers and the North American rivers (Figure 1; Table 1). The regions were defined based on their location, and physical and biological characteristics. Most of the data were collected in summer and δ^13^C‐POC_water_ did not vary seasonally (Appendix S1). In order to achieve the best spatial coverage, data from all seasons and years were combined for the spatial comparison. Regional means were calculated for δ^13^C‐POC_water_, δ^13^C‐POC_ice_, δ^13^C‐POC_riv_, δ^13^C‐DIC and δ^13^C‐CO_2_ values (Table 1).

**Table 1:** Location and description of marine regions and rivers, and regional means ± *SD* of δ^13^C values in dissolved inorganic carbon (DIC), ocean dissolved CO_2_, POC_water_, POC_ice_ and POC_riv_

Marine regions	Description	Regional mean ± *SD* (*n* = number of observations)				References
		δ^13^C‐DIC (‰)	δ^13^C‐CO_2_ (‰)	δ^13^C‐POC_water_ (‰)	δ^13^C‐POC_ice_ (‰)	
Outer shelves						
South Iceland	Atlantic influenced	1.3 ± 0.2 (*n* = 560)	−9.3 ± 0.2 (*n* = 560)	−19.9 ± 3.3 (*n* = 4)	NA	Becker et al. (2016); Key et al. (2015); Sarà et al. (2007); Schmittner et al. (2013); Young et al. ( 2013)
Norwegian sea	Atlantic influenced	1.4 ± 0.4 (*n* = 183)	−10.0 ± 0.4 (*n* = 183)	NA	NA	Bauch et al. (2015); Becker et al. (2016); Key et al. (2015)
Southeast Greenland	Atlantic influenced	1.3 ± 0.1 (*n* = 267)	−9.9 ± 0.2 (*n* = 267)	NA	NA	Becker et al. (2016); Key et al. (2015)
Hudson bay	Atlantic influenced; fresh water influenced	NA	NA	−24.7 ± 1.3 (*n* = 19)	NA	Kuzyk et al. (2010)
Bering sea	Pacific influenced	1.3 ± 0.6 (*n* = 11)	−9.8 ± 0.8 (*n* = 11)	−23.9 ± 0.7 (*n* = 62)	−21.5 ± 0.9 (*n* = 2)	Guo et al. (2004); Lin et al. (2014); Lovvorn et al. (2005); Schmittner et al. (2013); Smith et al. (2002); Young et al. (2013); Zhang et al. (2012)
Gulf of Alaska	Pacific influenced	0.8 ± 0.2 (*n* = 50)	−10.3 ± 0.3 (*n* = 50)	NA	NA	Schmittner et al. (2013); Young et al. (2013)
Inflow shelves						
Barents sea	Atlantic influenced	1.0 ± 0.4 (*n* = 10)	−10.3 ± 0.5 (*n* = 10)	−23.7 ± 1.6 (*n* = 12)	−19.3 ± 2.6 (*n* = 12)	Becker et al. (2016); Søreide et al. (2006); Tamelander et al. (2006, 2009)
Svalbard	Northwest of the Barents sea inflow shelf	1.3 ± 0.4 (*n* = 17)	−10.0 ± 0.4 (*n* = 17)	−24.5 ± 0.9 (*n* = 12)	−23.0 ± 0.7 (*n* = 6)	Becker et al. (2016); Søreide et al. (2006, 2008); Tamelander et al. (2009)
Svalbard fjords	Fresh water influenced; Northwest of the Barents sea inflow shelf	NA	NA	−26.5 ± 1.2 (*n* = 11)	NA	Hallanger et al. (2011); Kuliński et al. (2014)
Chukchi sea	Pacific influenced	0.8 ± 0.5 (*n* = 21)	−10.8 ± 0.7 (*n* = 21)	−22.7 ± 0.1 (*n* = 36)	NA	Bauch et al. (2015); Iken et al. (2010); Ivanov et al. (2012); Zhang et al. (2012)
Interior shelves						
Siberian coast	Fresh water influenced; consists of the East Siberian sea	NA	NA	−24.5 ± 0.5 (*n* = 7)	NA	Iken et al. (2010); Ivanov et al. (2012)
Beaufort sea	Fresh water influenced; North American coast	NA	NA	−26.7 ± 2.2 (*n* = 43)	−26.4 ± 0.5 (*n* = 8)	Connelly et al. (2015); Forest et al. (2010); Iken et al. (2005); O'Brien et al. (2006); Parsons et al. (1989); Zhang et al. (2012)
Outflow shelves						
Fram strait	Northeast of Greenland	1.3 ± 0.4 (*n* = 102)	−10.5 ± 0.4 (*n* = 102)	NA	NA	Bauch et al. (2015); Becker et al. (2016)
North‐East Water Polynia	Recurring mesoscale areas of open water within areas of pack ice (Sakshaug, 2004); Northeast of Greenland	NA	NA	−27.7 ± 0.6 (*n* = 3)	−18.6 ± 0.2 (*n* = 3)	Hobson et al. (1995)
North Water Polynia	Recurring mesoscale areas of open water within areas of pack ice (Sakshaug, 2004); North Baffin bay	NA	NA	−21.9 ± 0.6 (*n* = 30)	−17.7 ± 3.5 (*n* = 20)	Hobson et al. (2002); Tremblay et al. (2006)
Canadian archipelago	Complex straits and channels, terrestrial influence	NA	NA	−25.9 ± 1.4 (*n* = 21)	−18.9 ± 2.3 (*n* = 9)	Roy et al. (2015)
Arctic basins						
Arctic oceanic basins	Includes Amundsen, Nansen and Canadian basins	1.0 ± 0.2 (*n* = 134)	−11.1 ± 0.2 (*n* = 134)	−26.3 ± 1.6 (*n* = 88)	−22.1 ± 2.4 (*n* = 9)	Bauch et al. (2015); Brown et al. (2014); Griffith et al. (2012); Ivanov et al. (2012); Kohlbach et al. (2016); Schubert and Calvert (2001); Søreide et al. (2006); Tamelander et al. (2009); Zhang et al. (2012)

Riverine regions	Description			δ13C‐POC_riv_ (‰)		References
Siberian rivers	Includes the large rivers of Lena, Ob and Yenisei and smaller rivers of Kolyma, Indigirka, Yana, Olenek, Vakina and Mezen	NA	NA	−29.5 ± 2.1 (*n* = 237)	NA	Holmes et al. (2018); Lobbes et al. (2000)
North American rivers	Consists of the large rivers of Mackenzie and Yukon, as well as the smaller rivers surrounded the Hudson Bay (Figure 1)	NA	NA	−29.5 ± 1.7 (*n* = 146)	NA	Goni et al. (2000); Holmes et al. (2018); Kuzyk et al. (2010)

The decadal variation of regional marine δ^13^C‐POC_water_ values in arctic regions was assessed where data were available for at least three different years covering a period of at least 5 years. This included the following regions: arctic basins, Beaufort Sea, Chukchi Sea and Bering Sea. Svalbard and the Barents Sea, which had similar δ^13^C‐POC_water_ values and δ^13^C‐POC_ice_ values (Table S2: ANOVA3 and 4), were combined into the ‘European Arctic’ to achieve the best temporal coverage. The mean decadal trend (all regions combined) was calculated for δ^13^C‐POC_water_, δ^13^C‐POC_ice_, δ^13^C‐DIC and δ^13^C‐CO_2_ values.

### 2.3 Statistical analyses

Quantile–quantile plots of the residuals were plotted to check how closely the data follow a normal distribution (Becker, Chambers, & Wilks, 1988). The data were normally distributed, and therefore, we used a one‐way ANOVA (*α* = 0.005; Zuur, Ieno, & Smith, 2007) followed by post hoc Tukey pairwise comparison tests in R (R Core Team, 2018) to spatially compare: (a) the δ^13^C‐POC_water_ data between arctic shelves and arctic basins (ANOVA1), between arctic shelves and arctic rivers (ANOVA2) and between all arctic shelves (ANOVA3); and (b) the δ^13^C‐POC_ice_ values between all marine arctic regions where data were available (ANOVA4). We used a two‐way ANOVA followed by post hoc Tukey pairwise comparison test to compare the δ^13^C‐POC_ice_ values with δ^13^C‐POC_water_ values (factor ‘origin’) for regions (factor ‘region’) where both data sets were available (ANOVA5). Arctic regions with less than five data points were excluded from statistical analyses. Relevant *p*‐values of the post hoc Tukey pairwise comparison tests following ANOVA1 to 5 are shown in Table S2.

We applied linear models in R (R Core Team, 2018) to quantitatively assess the latitudinal gradient in δ^13^C‐DIC, δ^13^C‐CO_2_ and δ^13^C‐POC_water_ values, and the temporal trends in δ^13^C values of marine POC_water_, POC_ice_, DIC, dissolved CO_2_ and arctic marine mammals. The significance and robustness of the linear models were assessed based on the *p*‐values of the slopes and intercepts, the *R* ^2^, the *F*‐values and *df* (Table S3; Zuur et al., 2007).

## 3 RESULTS

### 3.1 Spatial trends in the δ^13^C of the baseline

**Figure 2:**
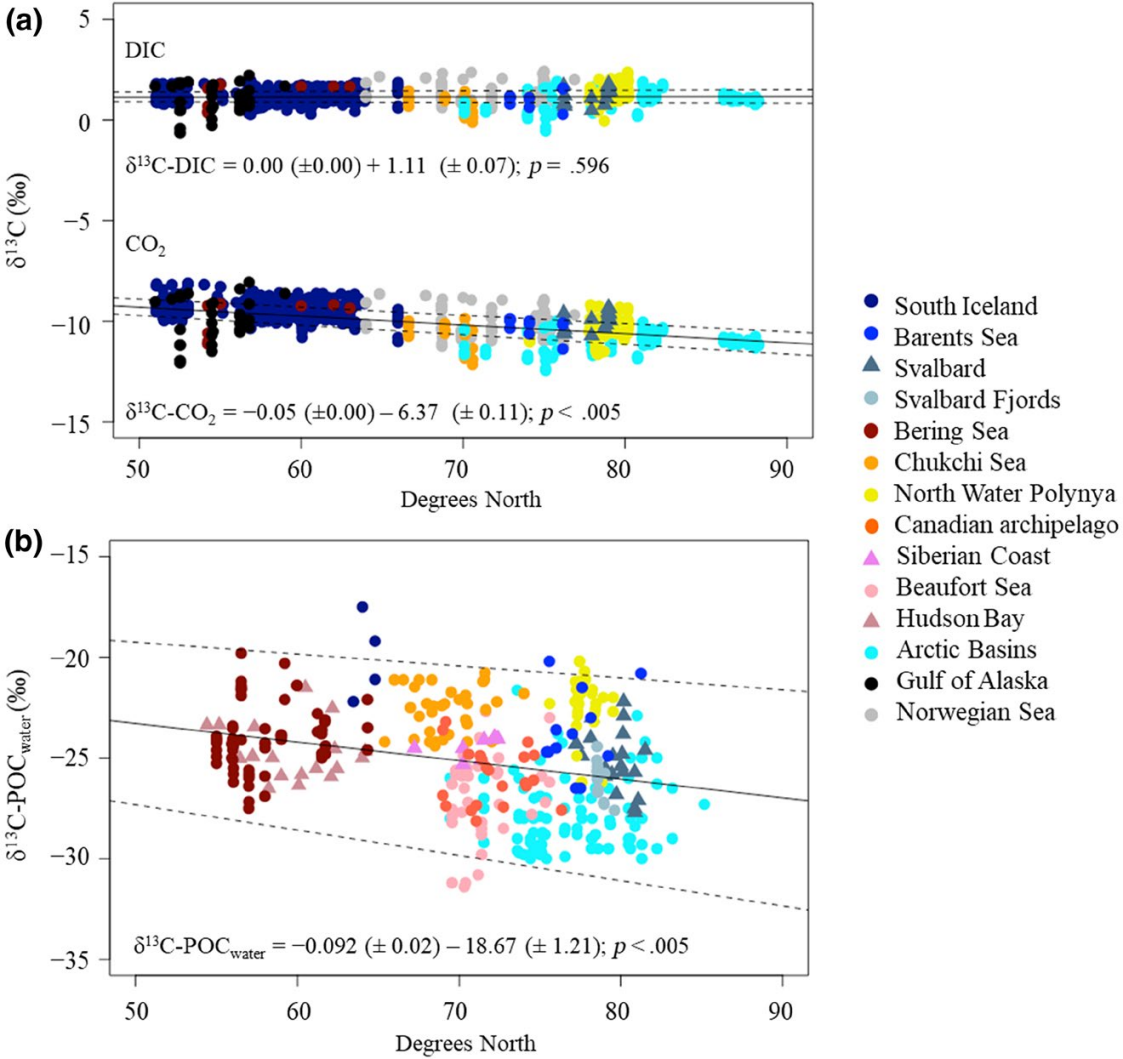
Stable carbon isotope values (δ^13^C, in ‰) of (a) marine dissolved inorganic carbon (DIC; *n* = 1,333) and marine dissolved CO_2_ (*n* = 1,333) and (b) marine POC_water_ (*n* = 354) in the surface waters with latitude; each dot is a single data point; the solid line represents the slope of the linear regression; dashed lines indicate the 95% confidence interval of the linear regression. The equations and *p*‐values of the linear regressions are shown on the figure. Trends are considered significant when *p* < .005

The Atlantic and Pacific waters entering the Arctic via the South Iceland and Norwegian Sea, and Gulf of Alaska and Bering Sea, respectively (Figure 1; Table 1), had similar δ^13^C‐CO_2_ values and were depleted by up to 2‰ relative to the δ^13^C‐CO_2_ values in the arctic basins (Table 1). We observed a significant depletion in δ^13^C‐CO_2_ and δ^13^C‐POC_water_ values with increasing latitude (Figure 2). δ^13^C‐DIC did not vary with latitude (Figure 2a).

**Figure 3:**
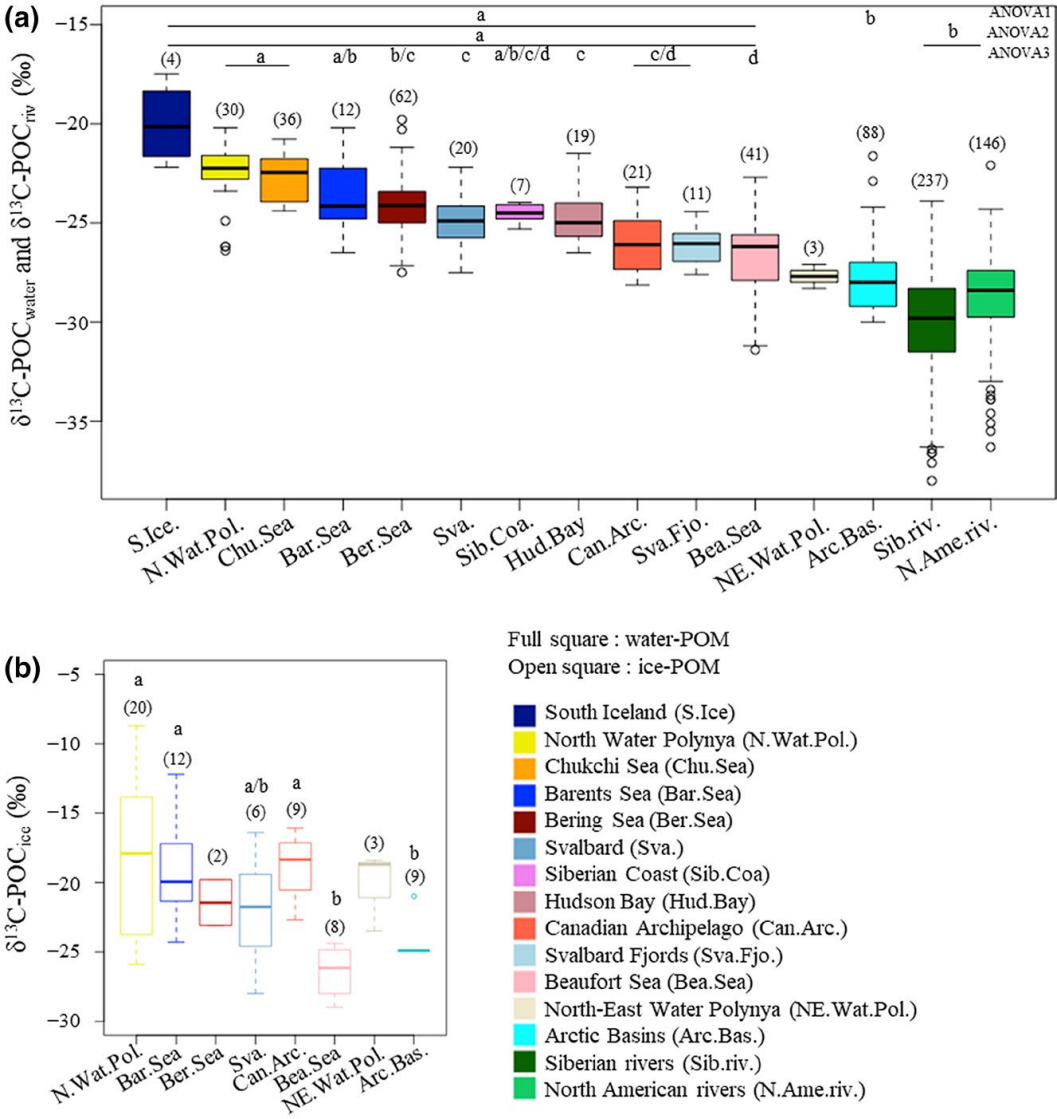
Regional stable carbon isotope values (δ^13^C, in ‰) of (a) POC_water_ and POC_riv_ and (b) POC_ice_; Numbers of observations are shown as number on top of the boxplots. Results of post hoc Tukey tests following (a) ANOVA1 to ANOVA3 and (b) ANOVA4 are expressed as letters on top of the boxplots. Different letters indicate significant differences (*p* < .005) between regions. The *p*‐values of each test are shown in Table S2

We analysed the δ^13^C‐POC_water_, δ^13^C‐POC_ice_ and δ^13^C‐CO_2_ values in 17 marine arctic regions (Figure 1; Table 1). δ^13^C values of POC_water_ varied significantly between arctic regions (Figure 3a). POC_water_ from arctic shelves was significantly enriched in ^13^C compared to POC_water_ from arctic basins and POC_riv_ (Figure 3a; Table S2: ANOVA1 and ANOVA2). The δ^13^C‐POC_water_ values were ^13^C depleted in arctic shelves (Beaufort Sea, Svalbard fjords, Canadian archipelago and the Hudson Bay) influenced by fresh water (Table 1; Figure 1) relative to the inflow (Chukchi Sea and Barents Sea) shelves and the North Water Polynya (Figure 3a; Table S2: ANOVA3).

δ^13^C‐POC_ice_ values followed the same regional trend as δ^13^C‐POC_water_ values, with δ^13^C‐POC_ice_ values enriched in ^13^C in the inflow and outflow shelves (Barents Sea, North Water Polynya) compared to the interior shelf Beaufort Sea and the arctic basins (Figure 3b; Table S2: ANOVA4).

### 3.2 Comparison between δ^13^C of POC_ice_ and POC_water_

Generally, δ^13^C values of POC_ice_ were significantly ^13^C‐enriched compared to those of POC_water_ (*p* < .005; Table S2: ANOVA5), with δ^13^C‐POC_water_ being enriched by 4.4‰ in the Barents Sea, by 4.2‰ in the North Water Polynya and by 7.0‰ in the Canadian archipelago (Table 1). There were no significant differences between POC_ice_ and POC_water_ in the Svalbard region, the arctic basins and the Beaufort Sea (Table S2: ANOVA5). δ^13^C‐POC_ice_ values were highly variable in most of the arctic regions (Figure 3b).

### 3.3 Temporal trends in the δ^13^C of the baseline and Arctic marine mammals

**Figure 4:**
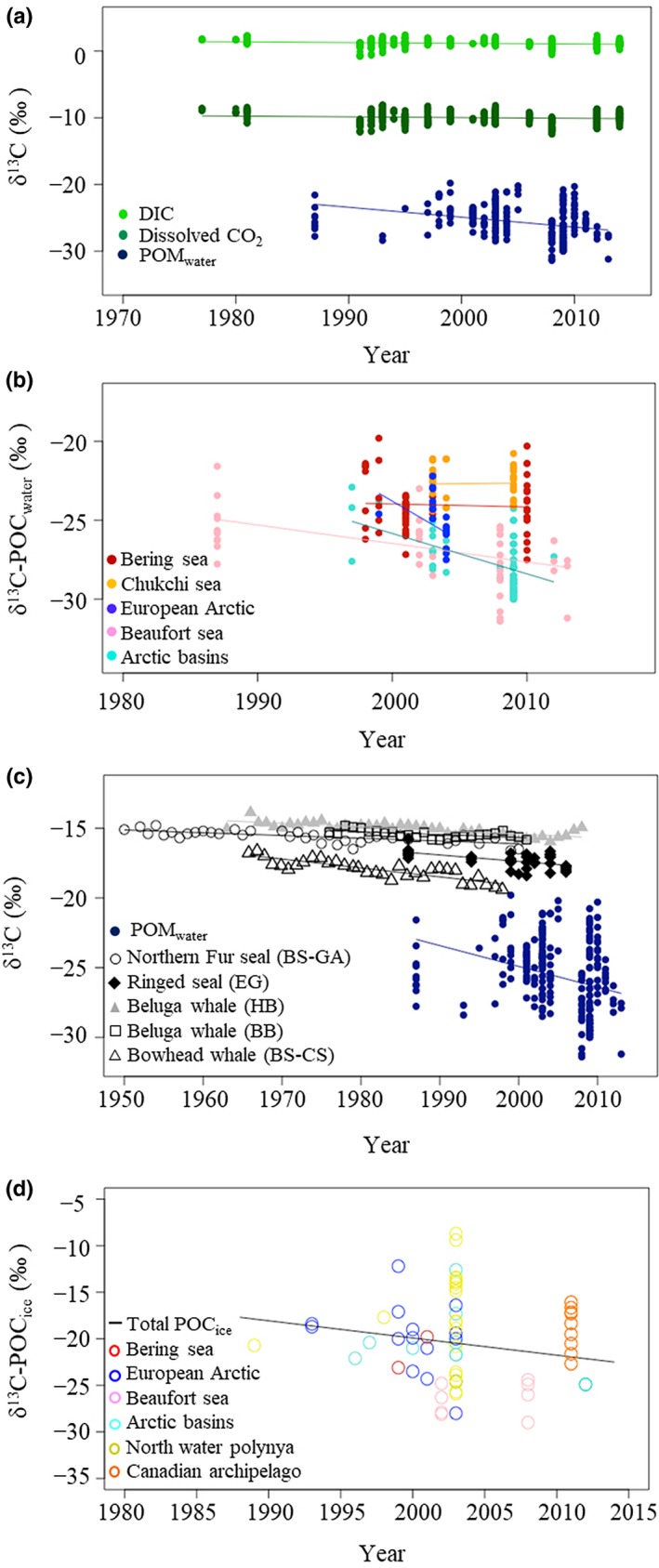
Decadal trend in δ^13^C values of: (a) dissolved inorganic carbon (DIC), dissolved CO_2_ and POC_water_, (b) POC_water_ for each arctic region, (c) POC_water_ and arctic marine mammal tissues and (d) POC_ice_ for each arctic region. BS, Bering sea; CS, Chukchi sea; EG, East Greenland; GA, Gulf of Alaska; HB, Hudson bay. Results of the linear models can be found in Table 2 and Table S3. Number of observations can be found in Table 2

In all arctic regions combined, δ^13^C‐DIC (1977–2014), δ^13^C‐CO_2_ (1977–2014) and δ^13^C‐POC_water_ (1986–2013) values became significantly ^13^C depleted by 0.011 ± 0.001, 0.011 ± 0.002 and 0.149 ± 0.020‰ per year respectively (Figure 4a; Table 2). The temporal trends in δ^13^C‐POC_water_ values were statistically significant in the Beaufort Sea (−0.117 ± 0.033‰ per year; 1987–2013) and in the arctic basins (−0.256 ± 0.057‰ per year; 1997–2012) and not statistically significant in the European Arctic, Bering Sea and Chukchi Sea (Figure 4b; Table 2; Table S3). The temporal trend in δ^13^C‐POC_ice_ values was not significant (Figure 4d; Table 2; Table S3). The δ^13^C values in the teeth of northern fur seals, ringed seals and beluga whales, and in baleen plates of bowhead whales were significantly depleted in ^13^C with time (Figure 4c; Table 2). The decline in δ^13^C values in teeth ranged from 0.020 ± 0.003‰ per year in northern fur seals from the Gulf of Alaska (1950–2000) to −0.046 ± 0.012‰ per year in ringed seals from East Greenland (1986–2006; Table 2). The δ^13^C in the baleen plates of bowhead whales from the Bering and Chukchi Seas significantly decreased by 0.064 ± 0.010‰ per year (1965–1998; Table 2). The decline in δ^13^C values of POC_water_ and marine mammals was larger than decline in δ^13^C‐DIC and δ^13^C‐CO_2_ values (0.011‰ per year, this study). Details of the linear models are shown in Table S3.

**Table 2:** Slopes ± *SD* and *p*‐values of the decadal linear models of δ^13^C values in dissolved inorganic carbon (DIC), ocean dissolved CO_2_, POC_water_, POC_ice_ and arctic marine mammal tissues

	Slope ± *SD*	*p*‐value	Time period	Number of observations
**POC_water_**				
**Beaufort sea**	−**0.117 ± 0.033**	**<.005**	**1987–2013**	**71**
European Arctic	−0.499 ± 0.265	.076	1999–2004	20
**Arctic basins**	−**0.256 ± 0.057**	**<.005**	**1997–2012**	**87**
Bering sea	−0.019 ± 0.046	.679	1998–2010	62
Chukchi sea	+0.008 ± 0.071	.906	2003–2009	36
**All data**	−**0.149 ± 0.020**	**<.005**	**1987–2013**	**311**
**POC_ice_**				
**All data**	−0.185 ± 0.106	.084	1993–2012	69
**DIC**				
**All data**	**−0.011 ± 0.001**	**<.005**	**1977–2014**	**1,333**
**CO_2_**				
**All data**	−**0.011 ± 0.002**	**<.005**	**1977– 2014**	**1,333**
**Marine mammals**				
**Northern fur seal—Bering sea/Gulf of Alaska**	**−0.020 ± 0.003**	**<.005**	**1950–2000**	**40**
**Ringed seal—East Greenland**	−**0.046 ± 0.012**	**<.005**	**1986–2006**	**36**
**Beluga whale—Hudson Bay**	**–0.026 ± 0.003**	**<.005**	**1963–2008**	**42**
**Beluga whale—Baffin Bay**	**–0.021 ± 0.006**	**<.005**	**1976–2001**	**26**
**Bowhead whale—Bering sea/Chukchi sea**	−**0.064 ± 0.007**	**<.005**	**1965–1998**	**34**

Note

Lines in bold are considered significant (*p* < .005).

Detailed statistics of the linear models are shown in Table S3.

## 4 DISCUSSION

### 4.1 Ice versus water

The ^13^C‐enrichment in POC_ice_ compared to POC_water_ in arctic regions has been observed previously and attributed to carbon limitation around ice algae within sea ice (Budge et al., 2008; Hobson et al., 2002; Søreide et al., 2006; Wang et al., 2014). The termination of the spring ice edge bloom can cause ^13^C at the base of the food web to be altered when ^13^C‐enriched ice algae were added to ^13^C‐depleted pelagic phytoplankton (Søreide et al., 2006). The similarity in the δ^13^C‐POC_ice_ and δ^13^C‐POC_water_ values in some regions (see Section 2.2) and the high intra‐regional variability of the δ^13^C‐POC_ice_ values may be explained by differences in ice porosity, allowing replenishment of DIC from water to ice (Thomas & Papadimitriou, 2011). δ^13^C‐POC_ice_ values were likely to have been influenced by light availability and the high bacterial activity in sea ice compared to open water (Wang et al., 2014). Thus, variation in the sampling month for sea ice might also contribute to the high variability in δ^13^C‐POC_ice_. This highlights that caution is required when using bulk δ^13^C values of POC_ice_ and POC_water_ to distinguish between open water versus ice‐dependent food webs in the Arctic (Søreide et al., 2006). The challenge of disentangling the contribution of carbon derived from sympagic production to the food web has been successfully resolved by using compound‐specific stable isotope analyses (e.g. δ^13^C values of fatty acids; Graham, Oxtoby, Wang, Budge, & Wooller, 2014; Oxtoby, Budge, Iken, Brien, & Wooller, 2016; Oxtoby et al., 2017; Wang et al., 2015).

### 4.2 Spatial trends

Spatial trends in the δ^13^C values of POC_water_ and POC_ice_ were similar, implying that they were influenced by the same environmental drivers within specific regions of the Arctic Ocean.

Low temperature, high wind speed and high productivity enhance the atmospheric CO_2_ uptake by the Arctic Ocean (Takahashi et al., 2002), driving strong latitudinal gradients in concentration and δ^13^C values of oceanic CO_2_ with ^13^C‐CO_2_ being more depleted in the Arctic Ocean (≈−10‰, Young et al., 2013; −10.2 ± 0.5‰, this study) relative to the tropics (≈−7 ‰, Young et al., 2013). In the marine environment, more than 90% of DIC is composed of bicarbonate ions (${\text{HCO}}_{3}^{-}$; Boutton, 1991). Fractionation between ${\text{HCO}}_{3}^{-}$ and atmospheric CO_2_ increases in cold water (Zhang, Quay, & Wilbur, 1995) leading to ^13^C enrichment of δ^13^C‐DIC values with increasing latitude (Tagliabue & Bopp, 2008), as observed in this study (Figure 2a). δ^13^C‐POC_water_ values became ^13^C‐depleted with increasing latitude (Figure 2b, this study; Goericke & Fry, 1994; McMahon et al., 2013b), reflecting the latitudinal trend in δ^13^C‐CO_2_ values as well as multiple additional factors, including temperature, phytoplankton growth rates, bacterial activity and isotopic fractionation, that also vary with latitude (Fouilland et al., 2018; Thomas, Kremer, Klausmeier, & Litchman, 2012; Young et al., 2013). A latitudinal trend in δ^13^C values of zooplankton was observed in the western Arctic (i.e. Bering and Chukchi Sea; Dunton, Saupe, Golikov, Schell, & Schonberg, 1989), demonstrating the transfer of this δ^13^C signature to the next trophic level.

The two orders of magnitude difference in phytoplankton production between the nutrient‐rich arctic shelves and the ice covered nutrient depleted arctic basin (Sakshaug, 2004) may partially explain the relatively large difference in δ^13^C‐POC_water_ values of 2.3‰ between the arctic shelf (−24.0 ± 1.2‰) and arctic basins (−26.3 ± 1.6‰). High rates of primary production cause ^13^C enrichment of the δ^13^C‐POC values (Boutton, 1991; McMahon, Hamady, & Thorrold, 2013a). The highly productive Bering Sea and Barents Sea account for up to two‐thirds of the total arctic phytoplankton production (Sakshaug, 2004). Advection of nutrients from the arctic outflow and early exposure to sunlight enhance phytoplankton productivity in the North Water Polynya (Sakshaug, 2004). In contrast, high turbidity and strong stratification caused by fresh water inflow from rivers onto the interior shelves reduce light and restrict phytoplankton production (Dittmar & Kattner, 2003). Lower phytoplankton productivity in the river influenced Beaufort Sea and Siberian Coast, as well as the North‐East Water Polynia (Sakshaug, 2004) could explain the depleted δ^13^C‐POC values observed in these regions relative to the more productive regions.

The ^13^C depletion in δ^13^C‐POC_water_ values observed in the interior shelves, Svalbard fjords, Hudson Bay and Canadian archipelago compared to other arctic shelf regions likely reflects the contribution of ^13^C‐depleted terrestrially derived POC (Boutton, 1991) from rivers, coastal erosion and glacial streams. Seventy‐two arctic rivers supplying 40% of the total freshwater input from the surrounding continents of Eurasia and North America enter the Arctic Ocean via the interior shelves of the Siberian coast and the Beaufort Sea (Table 1; Figure 1) at a rate of 2,500–4,200 km^3^/year (Haine et al., 2015). In addition, terrestrially derived POC input resulting from coastal erosion may be equal to or larger than input from river discharge in some regions, for instance along the Siberian coast (Rachold et al., 2000). Finally, glacial fjords on Svalbard are fed with freshwater by large glaciers and streams with the highest freshwater inflow in summer during ice and snow melt (Cottier et al., 2005). Any temporal alteration of the riverine inputs or the drainage basins would likely alter the δ^13^C‐POC_water_ values in the interior shelves and subsequently alter the base of the food web.

### 4.3 Temporal trends at the baseline

The increasing concentration of anthropogenic CO_2_, known as the Suess effect, is predicted to decrease the oceanic δ^13^C‐DIC values by an average of 0.017‰ per year, with high spatial variability from 0‰ per year in the Southern Ocean to 0.024‰ per year in the subtropical gyres (Tagliabue & Bopp, 2008). In the Arctic Ocean, the δ^13^C‐DIC values are predicted to decrease by 0.006‰ to 0.008‰ per year (Tagliabue & Bopp, 2008). We observed a decreasing trend in δ^13^C‐DIC values of 0.011 ± 0.001‰ per year from 1977 to 2014 across all arctic regions, which is larger than the predicted trend. Although CO_2_ represents less than 0.5% of the total DIC pool, it is the only component that is exchangeable with the atmosphere. In polar regions, especially the Arctic Ocean, the decline in sea ice has led to an expansion of open water (Arrigo & van Dijken, 2015). This facilitates atmospheric exchange and enhances the dissolved CO_2_ concentration (Yamamoto et al., 2012) resulting in an additional ^13^C depletion of δ^13^C‐CO_2_ values (Rau et al., 1992) which may explain the larger decrease in δ^13^C‐CO_2_ values (0.011 ± 0.002‰ per year) and in turn the larger decrease in δ^13^C‐DIC values (0.011 ± 0.001‰ per year) in the Arctic Ocean compared to the predicted decrease of 0.006–0.008‰ per year (Tagliabue & Bopp, 2008).

The decadal decline in δ^13^C‐POC_water_ values (1987–2013) was more than 10 times larger than the trend in δ^13^C values of CO_2_ (or DIC) implying that other factors are influencing the δ^13^C values in POC in the Arctic Ocean. Since the mid‐1990s, sea ice extent has declined by 8.3 ± 0.6% per decade across the entire Arctic (Comiso, 2012). Sea ice algae are up to 7 ‰ enriched in ^13^C relative to pelagic phytoplankton (this study) and a decline in sea ice could decrease the contribution of ice algal biomass to total productivity and reduce the total mean δ^13^C values of POC_water_. For example, the open water area of the Barents sea has increased by 15,789 km^2^ or 1.3% per year between 1998 and 2012, alongside a 28% increase in net primary production over the same time period (Arrigo & van Dijken, 2015). Assuming distinct end members for δ^13^C‐POC_water_ (−25.0 ± 1.7‰) and δ^13^C‐POC_ice_ (−20.0 ± 1.3‰) values, sea ice decline would cause the entire pool of δ^13^C‐POC values to decrease by 0.06 ± 0.15‰ per year. Additionally, the photosynthetic isotopic fractionation factor for phytoplankton in the Arctic Ocean has increased by 0.045‰ per year since the 1960s, compared to a global average of 0.022‰ per year (Young et al., 2013). The combined effect of a decline in ice algae (0.06 ± 0.15‰ per year, this study), increase in fractionation factor (0.045‰ per year, Young et al., 2013) and Suess effect (i.e. dissolved CO_2_, 0.011 ± 0.001‰ per year, this study) could potentially cause the δ^13^C‐POC values to decrease by 0.116 ± 0.15‰ per year, which is of the same order of magnitude as the observed annual decrease in δ^13^C‐POC_water_ values in the whole Arctic (0.149 ± 0.028‰ per year) and in the Beaufort Sea (0.126 ± 0.020‰ per year; Table 2). In support of this argument, the difference between the temporal trend or slope in δ^13^C‐CO_2_ and δ^13^C‐POC values (Figure 4a) increased by 0.138 ± 0.028‰ per year in agreement with the sum of the contributions from a change in ice (0.06 ± 0.15‰ per year), fractionation (0.045‰ per year) and Suess effect (0.011 ± 0.001‰ per year) influencing δ^13^C‐POC_water_ values.

Other factors contributing to the decline in δ^13^C‐POC values in the Arctic Ocean include river run‐off, coastal erosion, primary production and bacterial activity. Increased riverine run‐off (Haine et al., 2015) and coastal erosion (Jones et al., 2009; Mars & Houseknecht, 2007) resulting from ongoing climate change in the Arctic could contribute to the decline in δ^13^C‐POC values by adding ^13^C‐deplete terrestrially derived material to the marine POC pool. Changes in primary productivity will also influence the δ^13^C‐POC values. For example, the decline of δ^13^C values in Bowhead whales from the Bering/Chukchi Sea was interpreted by Schell (2000) as reflecting a 30%–40% decrease in seasonal primary productivity in the Bering Sea over the last 30 years. Increasing bacterial activity with increasing temperature (Vaqué et al., 2019; Vernet, Richardson, Metfies, Nöthig, & Peeken, 2017) and dissolved CO_2_ concentration (Grossart, Allgaier, Passow, & Riebesell, 2006) in the Arctic may also influence the δ^13^C values of POC.

### 4.4 Implications for food web

The reliability of stable carbon isotopes in deciphering the provenance of feeding or migratory patterns of consumers is heavily dependent on knowledge of δ^13^C values at the base of the food web. Maps that convey the geographical and temporal trends of δ^13^C values in the baseline, termed isoscapes (Bowen et al., 2009; Graham et al., 2010), have become a necessity for interpreting trophic structure using δ^13^C (or δ^15^N) values (Hansen, Hedeholm, Sünksen, Christensen, & Grønkjær, 2012; Newsome, Clementz, & Koch, 2010). Although isoscapes have been constructed for the atmosphere (Bowen et al., 2009), terrestrial environment (Bowen & West, 2008; Firmin, 2016) and the Atlantic and Pacific Oceans (Graham et al., 2010; McMahon et al., 2013b), this study provides a first view of δ^13^C‐POC values or carbon isoscape of the Arctic Ocean. We found spatially heterogeneous and temporally evolving δ^13^C values in the POC pool, which has ramifications for the study of food webs in space and time.

Previous studies have noted that the decline in δ^13^C in Arctic marine mammals is larger than the Suess effect alone (e.g. Matthews & Ferguson, 2014; Newsome et al., 2007), but the lack of δ^13^C baseline information prevented these authors from disentangling the driving factors (Cullen, Rosenthal, & Falkowski, 2001; Schell, 2000, 2001). Generally, the temporal decline in the δ^13^C values in marine mammals was larger than in δ^13^C‐DIC and δ^13^C‐CO_2_ values (both of −0.011 ± 0.001‰ per year) but smaller than the decline observed in δ^13^C‐POC_water_ values (−0.149 ± 0.028‰ per year). The δ^13^C signature in phytoplankton or a consumer represents an average ratio related to the lifetime of the organism and tissue turnover time (Vander Zanden, Clayton, Moody, Solomon, & Weidel, 2015). Previous studies have shown that the seasonal variation in δ^13^C values of POC was higher than in higher trophic levels reflecting the strong seasonal growth cycle of phytoplankton and shorter time period over which they integrate carbon (O'reilly, Hecky, Cohen, & Plisnier, 2002). In contrast, consumers from zooplankton to predators are long‐lived and thus integrate δ^13^C values over their seasonal foraging and migratory routes (Aubail et al., 2010; Schell, Saupe, & Haubenstock, 1989) with the time of integration depending on the tissue type (Vander Zanden et al., 2015) or the animals’ lifetime (O'reilly et al., 2002). The effect of yearly averaging of the δ^13^C values in marine mammal teeth and baleen plates used to reconstruct decadal trends may have reduced the larger, short‐lived variation observed in δ^13^C‐POC values mainly representing summer in this study. The gradual linear decline in δ^13^C values in arctic seals and whales likely reflects alterations to the δ^13^C‐POC values. A change in diet, for example, a shift towards foraging closer to freshwater (Nelson et al., 2018), or more pelagic feeding habits (Aubail et al., 2010), may also contribute to the temporal decline in δ^13^C values observed in predators.

This study demonstrates that to disentangle factors driving variation in the δ^13^C values in a consumer, it is vital to know the spatial heterogeneity and temporal evolution of δ^13^C values of the baseline in the Arctic Ocean in order to avoid inaccurate interpretation of changes in food web structures. Some studies have attempted to correct the δ^13^C values in arctic marine mammals for the Suess effect using modelled and predicted values for large geographical regions, prior to interpreting decadal trends in δ^13^C values (Carroll, Horstmann‐Dehn, & Norcross, 2013; Misarti et al., 2009; Nelson et al., 2018). However, the Suess effect varies spatially (Tagliabue & Bopp, 2008), and therefore, local values should be used for this correction. For example, the Suess effect in the Arctic Ocean (0.011 ± 0.001‰ per year, this study) differs from the predicted modelled values (0.006–0.008‰ per year; Tagliabue & Bopp, 2008), implying that other factors, such as the loss of sea ice, are accelerating the influence of anthropogenic CO_2_ in the Arctic. In addition, the decline in δ^13^C‐POC values, representing the base of the food web, is larger than the decline in δ^13^C‐DIC values (this study). This suggests that interpretation about diet shift should be done after consideration of temporal trends in δ^13^C‐POC values and not only in δ^13^C‐DIC (Suess effect). These results also highlight the importance of considering time‐averaging effects when studying different trophic levels and/or tissues having, respectively, variable lifetime and turnover time. Insight from this study has direct implications for how we interpret changes in δ^13^C values in consumers, especially in environments experiencing rapid change.
