# Supplementary material for: Temporal and spatial trends in marine carbon isotopes in the Arctic Ocean and implications for food web studies
Source: Glob Chang Biol. 2019 Oct 10;25(12):4116–30. doi: 10.1111/gcb.14832 (PMC6899536; doi:10.1111/gcb.14832)
Supplement: Supplementary file 1 [file GCB-25-4116-s001.pdf]

Appendix S1: Seasonal variation

δ^{13}C-POC$_{water}$ can vary seasonally as a result of variation in phytoplankton growth and respiration rates (Boutton 1991). Seasonal variation of marine δ^{13}C-POC$_{water}$ was assessed for regions where data was available for at least two seasons (e.g. spring and summer separated by maximum of five years). This includes: the Barents Sea from 2001 to 2005, the Svalbard in 2003 and 2004, the Beaufort Sea from 2008 to 2012, the Bering Sea from 1999 to 2003 and the North Water Polynya in 2003.

A two-way analysis of variance (ANOVA) was applied for the factors region and season followed by post hoc Tukey pairwise comparison tests. δ^{13}C-POC$_{water}$ did not significantly vary between spring and summer within each considered Arctic regions (Table S1).

Table S1: Results of the two-way ANOVA applied for the factors region and season and the relevant post hoc Tukey pairwise comparison tests

**ANOVA**	p-values
ANOVA factor 'season'	0.017
ANOVA factor 'region'	$< 2x10^{-16}$
**post hoc Tukey tests**	
Barents Sea: spring *versus* summer	0.050
Svalbard: spring *versus* summer	0.162
Beaufort Sea: spring *versus* summer	0.390
Bering Sea: spring *versus* summer	0.999
North Water Polynya: spring *versus* summer	0.443

Regional differences in δ^{13}C-POC$_{water}$ (ANOVA factor ‘region’; Table S1) are larger than the seasonal differences (ANOVA factor ‘season’; Table S1).
