# Supplementary material for: Temporal and spatial trends in marine carbon isotopes in the Arctic Ocean and implications for food web studies
Source: Glob Chang Biol. 2019 Oct 10;25(12):4116–30. doi: 10.1111/gcb.14832 (PMC6899536; doi:10.1111/gcb.14832)
Supplement: Supplementary file 2 [file GCB-25-4116-s002.pdf]

S2: Relevant p-values from the post hoc Tukey comparison tests following one-way ANOVA1: spatial comparison between $\delta^{13}C$-POC_{water} data from arctic shelves and arctic basins; one-way ANOVA2: spatial comparison between $\delta^{13}C$-POC_{water} data from arctic shelves and arctic rivers; one-way ANOVA3: spatial comparison between $\delta^{13}C$-POC_{water} data from arctic shelves; one-way ANOVA4: spatial comparison of the $\delta^{13}C$-POC_{ice} data; two ways ANOVA5 including the factors "POC origin" (ice *versus* water) and "arctic region": comparisons between $\delta^{13}C$-POC_{ice} with $\delta^{13}C$-POC_{water} for each arctic regions.

ANOVA1 (POC_{water})	Arctic basins
South Iceland	<0.005
North water polynya	<0.005
Chukchi sea	<0.005
Barents sea	<0.005
Bering sea	<0.005
Svalbard	<0.005
Siberian coast	<0.005
Hudson bay	<0.005
Svalbard fjords	0.045
Beaufort sea	<0.005
Canadian archipelago	<0.005

ANOVA2 (POC_{water}; POC_{riv})	Siberian rivers	North American rivers
South Iceland	<0.005	<0.005
North water polynya	<0.005	<0.005
Chukchi sea	<0.005	<0.005
Barents sea	<0.005	<0.005
Bering sea	<0.005	<0.005
Svalbard	<0.005	<0.005
Siberian coast	<0.005	<0.005
Hudson bay	<0.005	<0.005
Svalbard fjords	<0.005	<0.005
Beaufort sea	<0.005	<0.005
Canadian archipelago	<0.005	<0.005

ANOVA3 (POC_{water})	Chukchi sea	Barents sea	Bering sea	Svalbard	Siberian coast	Hudson bay	Svalbard fjords	Beaufort sea	Canadian archipelago
North water polynya	0.999	0.420	<0.005	<0.005	0.084	<0.005	<0.005	<0.005	<0.005
Chukchi sea	-	0.618	<0.005	<0.005	0.149	<0.005	<0.005	<0.005	<0.005
Barents sea	-	-	0.999	0.518	0.989	0.792	0.011	<0.005	0.007
Bering sea	-	-	-	0.383	0.999	0.798	<0.005	<0.005	<0.005
Svalbard	-	-	-	-	0.999	0.999	0.586	<0.005	0.651
Siberian coast	-	-	-	-	-	0.999	0.495	0.019	0.584
Hudson bay	-	-	-	-	-	-	0.343	<0.005	0.358
Svalbard fjords	-	-	-	-	-	-	-	0.976	0.999
Beaufort sea	-	-	-	-	-	-	-	-	0.557

ANOVA4 (POC_{ice})	Svalbard	Amundsen-Nansen basins	North water polynya	Beaufort sea	Canadian archipelago
Barents sea	0.484	0.006	1.000	<0.005	0.999
Svalbard	-	0.789	0.352	0.071	0.381
Amundsen-Nansen basins	-	-	<0.005	0.519	0.006
North water polynya	-	-	-	<0.005	0.999
Beaufort Sea	-	-	-	-	<0.005

ANOVA5	Water *versus* ice
Barents sea	0.134
Svalbard	0.527
Amundsen-Nansen basins	<0.005
North water polynya	0.999
Beaufort sea	0.999
Canadian archipelago	<0.005
