# Supplementary material for: Temporal and spatial trends in marine carbon isotopes in the Arctic Ocean and implications for food web studies
Source: Glob Chang Biol. 2019 Oct 10;25(12):4116–30. doi: 10.1111/gcb.14832 (PMC6899536; doi:10.1111/gcb.14832)
Supplement: Supplementary file 3 [file GCB-25-4116-s003.pdf]

S3: Slopes, intercepts, and statistics of the decadal linear models of $\delta^{13}C$ in ocean dissolved CO_2, dissolved inorganic carbon (DIC), POC_{water}, POC_{ice} and arctic marine mammal tissues; DF = degrees of freedom; Lines in bold are considered significant ($p < 0.005$).

	Slope			Intercept			Linear model	
	slope	SD	p-value	Intercept	SD	p-value	R^2 (%)	F-statistic (DF)
**POC_{water}**								
**Beaufort sea**	**–0.117**	**–0.033**	**<0.005**	**207.7**	**67.1**	**<0.005**	**21.1**	**12.22 (41)**
European Arctic	–0.499	0.265	0.076	974.2	530.7	0.08	42.9	3.5 (18)
**Arctic basins**	**–0.256**	**–0.057**	**<0.005**	**486.4**	**114.3**	**<0.005**	**19.2**	**20.2 (85)**
Bering sea	–0.019	0.046	0.679	14.7	92.9	0.875	0.29	0.19 (60)
Chukchi sea	+0.008	0.071	0.906	–39.6	141.9	0.782	0.85	0.01 (34)
**All data**	**–0.149**	**0.028**	**<0.005**	**273.0**	**55.2**	**<0.005**	**9.4**	**29.3 (311)**
POC_{ice}								
All data	–0.185	0.106	0.084	350.5	211.9	0.103	2.9	3.1 (69)
**DIC**								
**All data**	**–0.011**	**0.001**	**<0.005**	**22.8**	**2.5**	**<0.005**	**5.1**	**72.9 (1333)**
**CO_2**								
**All data**	**–0.011**	**0.002**	**<0.005**	**12.1**	**4.9**	**0.013**	**1.5**	**20 (1333)**
**Marine mammals**								
**Northern fur seal - Bering sea/Gulf of Alaska**	**–0.020**	**0.003**	**<0.005**	**23.9**	**6.5**	**<0.005**	**49.2**	**36.8 (38)**
**Ringed seal - East Greenland**	**–0.046**	**0.012**	**<0.005**	**74.7**	**23.4**	**<0.005**	**31.3**	**15.5 (34)**
**Beluga whale –Hudson Bay**	**–0.026**	**0.003**	**<0.005**	**36.6**	**6.7**	**<0.005**	**57.9**	**60.1 (38)**
**Beluga whale – Baffin Bay**	**–0.021**	**0.006**	**<0.005**	**25.7**	**11.8**	**0.040**	**30.8**	**12 (24)**
**Bowhead whale – Bering sea/Chukchi sea**	**–0.064**	**0.007**	**<0.005**	**104.2**	**13.6**	**<0.005**	**72.3**	**80.7 (32)**
